# The Association of Periodontitis With Risk of Prevalent and Incident Metabolic Syndrome

**DOI:** 10.1111/jcpe.70042

**Published:** 2025-09-23

**Authors:** Jenni Kinnunen, Kari Koponen, Oleg Kambur, Muhammed Manzoor, Katariina Aarnisalo, Verneri Nissilä, Satu Männistö, Veikko Salomaa, Pekka Jousilahti, Eija Könönen, Ulvi Kahraman Gürsoy, Aki S. Havulinna, Aino Salminen, Pirkko Pussinen

**Affiliations:** ^1^ School of Medicine, Institute of Dentistry University of Eastern Finland Kuopio Finland; ^2^ Department of Public Health Finnish Institute for Health and Welfare Helsinki Finland; ^3^ Department of Bacteriology and Immunology University of Helsinki Helsinki Finland; ^4^ Oral and Maxillofacial Diseases University of Helsinki and Helsinki University Hospital Helsinki Finland; ^5^ Periodontology, Institute of Dentistry University of Turku Turku Finland; ^6^ Institute for Molecular Medicine Finland, FIMM‐HiLIFE University of Helsinki Helsinki Finland; ^7^ Department of Computing University of Turku Turku Finland

**Keywords:** biomarkers, metabolic syndrome, obesity, periodontitis, saliva

## Abstract

**Aim:**

To investigate whether periodontitis is associated with prevalent and incident metabolic syndrome (MetS).

**Materials and Methods:**

The baseline study included 4183 individuals from a population‐based survey (DILGOM) in 2007 and follow‐up of 1047 participants with clinical re‐examination in 2014. The risk of periodontitis was assessed with saliva biomarkers using a validated, three‐group cumulative risk score for periodontitis (CRS I, II and III).

**Results:**

In fully adjusted models, CRS III was associated with prevalent MetS (OR: 1.35, 95% CI [1.11–1.65]), high waist circumference (1.55 95% CI [1.26–1.91]), high blood pressure (1.29 95% CI [1.05–1.59]) and the number of MetS components (β: 0.18, 95% CI [0.06–0.30]). Among participants without MetS at baseline (*n* = 618), 128 (20.7%) developed MetS during follow‐up. In the fully adjusted model, CRS III trended positively with incident MetS (RR: 1.55, 95% CI [ 0.96–2.51]) in the whole population and had a significant positive association in women (2.06, 95% CI [1.08–3.94]), and in non‐smokers (1.78, 95% CI [1.01–3.14]). The risk between CRS and incident MetS was mediated via systemic inflammation.

**Conclusion:**

Periodontitis is associated with an increased risk of having metabolic syndrome and, in particular, clearly with the number of MetS components: abdominal obesity, hyperglycaemia and hypertension. Systemic inflammation may elucidate the observed higher risk of incident MetS.

## 1 Introduction

Periodontitis is a common dysbiosis‐driven chronic inflammatory disease affecting tooth‐attachment tissues. It is diagnosed during clinical and radiographic oral examinations. However, these are laborious in large‐scale studies, where the use of biomarkers would be more feasible. The cumulative risk score (CRS) for periodontitis consists of three salivary biomarkers: *Porphyromonas gingivalis* , interleukin (IL)‐1β and matrix metalloproteinase (MMP)‐8 (Gürsoy et al. 2011), with IL‐1β reflecting inflammation, MMP‐8 tissue destruction and *P. gingivalis* periodontal pathogen burden. These biomarkers have been shown to distinguish patients with periodontitis from periodontally healthy individuals by classifying participants into low‐, moderate‐ and high‐risk groups (Gürsoy et al. 2011; Salminen et al. 2014). The biomarkers are also associated with the subgingival burden of Gram‐negative bacterial species, saliva lipopolysaccharide (LPS) activity and antibody levels against periodontitis‐associated bacterial species (Liukkonen et al. 2020). Although the CRS has been validated with clinical and radiographic periodontal features, and its association with local bacterial species and the host response against them have been shown, its relation to systemic conditions has not yet been studied.

Metabolic syndrome (MetS) is a condition characterised by the simultaneous presence of several health‐threatening metabolic disorders (Alberti et al. 2009). Insulin resistance and central obesity are considered the most important risk factors for the development of MetS, whereas other risk factors include lifestyle characteristics such as physical inactivity and smoking, as well as genomics and ageing (McCracken et al. 2018; Pirih et al. 2021). The pathogenesis of MetS is not fully understood but may involve insulin resistance induced by fatty acid dysfunction, low‐grade chronic inflammation and oxidative stress (McCracken et al. 2018). MetS increases the risk of developing cardiovascular diseases and type 2 diabetes (McCracken et al. 2018; Pirih et al. 2021).

Several studies have investigated the association between periodontitis and MetS across different populations (Adachi and Kobayashi 2020; Ayuthaya et al. 2024; Campos et al. 2022; Daudt et al. 2018; Morita et al. 2010; Pirih et al. 2021; Rosário‐Dos‐Santos et al. 2023; Saito et al. 2024; Sakurai et al. 2019; Yan et al. 2024). Findings from previous studies have concluded that the risk of MetS is associated with the severity of periodontitis, with the summary odds ratio (OR) of 1.26 (2.10–5.37) in moderate periodontitis and 1.50 (1.28–1.17) in severe cases (Rosário‐Dos‐Santos et al. 2023). A recent systematic review including 51 studies, of which 38 were used in a meta‐analysis, supports a positive association between MetS and periodontitis in a dose‐dependent manner (Campos et al. 2022). The underlying mechanisms are not fully understood, but dysbiosis and host response, including chronic systemic inflammation, may play a role (Campos et al. 2022).

Most of the published studies are cross‐sectional, and longitudinal research remains scarce. Among five studies conducted among Asian populations, four (Ayuthaya et al. 2024; Morita et al. 2010; Saito et al. 2024; Sakurai et al. 2019) identified a positive association between periodontitis and incident MetS, conversion of MetS components or increased number of MetS components, while one (Adachi and Kobayashi 2020) did not support a link. All previous studies are based on clinical oral examination. Thus, our aim was to investigate the association between periodontitis—defined by salivary biomarkers as a novel tool—and prevalent and incident MetS in a population‐based cohort.

## 2 Materials and Methods

### 2.1 Study Population

DIetary, Lifestyle and Genetic determinants of Obesity and Metabolic syndrome (DILGOM) is an expansion of the FINRISK 2007 survey, where all participants were invited to an extensive clinical examination (*n* = 5024). The recruitment protocol and details have been reported earlier (Kanerva et al. 2018). Eligible participants of the baseline DILGOM with complete data were invited to take part in the DILGOM 2014 follow‐up phase. One‐thousand three‐hundred and twelve participants underwent a similar clinical examination as conducted during the baseline phase and filled in a health‐related questionnaire. The included clinical laboratory investigations enabled setting the MetS diagnosis. These participants comprised the follow‐up group of the present study. Details of the study population are described in Supporting Information, and a flow chart clarifying the study design is shown in Figure S1.

### 2.2 Metabolic Syndrome Definition

Participants with MetS were categorised using the International Diabetes Federation (IDF) criteria. Medications were classified according to the Anatomical Therapeutic Chemical (ATC) classification system. The data on medications was obtained from the National Drug Reimbursement Register.

We set the MetS diagnoses according to the updated criteria, when an individual had (1) elevated waist circumference (≥ 94 cm for men and ≥ 80 cm for women, European‐origin‐specific definition) in combination with any two of the following factors: (2) elevated blood pressure (systolic blood pressure ≥ 130 mmHg and/or diastolic blood pressure ≥ 85 mmHg or treatment of previously diagnosed hypertension [ATC code C02, antihypertensives]); (3) elevated fasting plasma glucose concentration (fasting glucose level ≥ 5.6 mmol/L or type 2 diabetes and treatment [ATC code A10, drugs used in diabetes]); (4) elevated triglyceride concentration (triglyceride level ≥ 1.7 mmol/L or cholesterol‐lowering medication [ATC codes C10A, C10B, lipid‐modifying agents]); or (5) reduced high‐density lipoprotein (HDL) cholesterol concentration (HDL cholesterol < 1.0 mmol/L in males and < 1.3 mmol/L in females or cholesterol medication [ATC codes C10A, C10B]) (Alberti et al. 2009; WHO Collaborating Centre for Drug Statistics Methodology 2023).

### 2.3 Saliva Samples and Biomarker Quantification

Paraffin‐stimulated saliva samples were collected from participants of the DILGOM 2007 study and frozen at −70°C until laboratory analyses. DNA was extracted using a Chemagic 360 instrument (Perkin Elmer). *P. gingivalis* was determined by quantitative real‐time PCR (qPCR) assay, and qPCR reactions were performed with a QuantStudio5 real‐time PCR instrument (Thermo Fisher, Singapore) using the KAPA SYBR FAST universal master mix (Merck SA, Darmstadt, Germany) (Hyvärinen et al. 2009). Quantification of matrix metalloproteinase‐8 (MMP‐8) was done using ELISA (Elabscience, E‐EL‐H1450) on the DS2 automated ELISA system (Dynex Technologies Ltd., West Sussex, UK), and interleukin‐1β using Luminex technology (Bio‐Rad, Bio‐Plex Pro Human Cytokine Th17 15‐plex). DNA was successfully isolated and biomarkers were determined from a total of 4183 (83%) samples.

### 2.4 Periodontal Status and Cumulative Risk Score (CRS)

Periodontal status was assessed using salivary biomarker concentrations to form the CRS, a diagnostic tool that classifies individuals into three risk groups for having periodontitis (Gürsoy et al. 2011). In this score, salivary concentrations of IL‐1β (pg/mL) and MMP‐8 (ng/mL) were divided into tertiles, giving each tertile a value of 1, 2 or 3, respectively. The cut‐off points were 39.23 and 67.72 pg/mL for IL‐1β, and 398.14 and 780.69 ng/mL for MMP‐8. Instead of tertiles, *P. gingivalis* concentrations were divided into three groups, defined as 0 genomes/mL (value 1), below median (value 2) and above median (value 3). The median value of *P. gingivalis* concentrations was 2,010,524 genomes/mL after excluding those individuals whose measurement was 0 (*n* = 3437). An individual CRS was calculated by multiplying the acquired values of the three biomarkers, yielding possible scores of 1, 2, 3, 4, 6, 8, 9, 12, 18 or 27. Based on this score, participants were categorised into the following groups: CRS I (lowest risk of having periodontitis; scores 1, 2), CRS II (medium risk; scores 3, 4, 6, 8) and CRS III (highest risk; scores 9, 12, 18, 27) (Gürsoy et al. 2011; Salminen et al. 2014).

### 2.5 Statistical Analysis

Statistical analyses were performed with R software (version 4.2.2) (R Core Team 2022). Differences in means between groups were assessed using Welch's *t*‐test for unequal variances for comparisons between two groups and one‐way ANOVA for comparisons between three groups. Differences in proportions of categorical variables were analysed using Pearson's Chi‐squared test. Associations between cross‐sectional (cases at baseline) and follow‐up (new cases detected in the follow‐up health examination visit) MetS and CRS groups were analysed using binary logistic regression and modified Poisson regression, respectively. Mediation between CRS (modelled as a continuous variable) and MetS was checked via systemic inflammation (C‐reactive protein, CRP) and insulin resistance (Homeostatic Model Assessment of Insulin Resistance, HOMA‐IR). Mediation analysis was performed using the PROCESS tool (Hayes 2022), using binary logistic regression for the final assessment for direct and indirect effects. Bootstrapped mean coefficients were reported with the number of bootstrap iterations set at 10,000. Regression models were run and reported using the following adjustment configurations: Model 1 was adjusted for demographic covariates (baseline age and sex), and Model 2 included baseline age, sex, smoking status and the number of years of education. Additionally, we assessed diet, physical activity, household income and an interaction effect between baseline age and sex in Model 3 (full adjustment plus an age–sex interaction term). Stratified sensitivity analyses were run separately for sex, smoking status and 10‐year age groups. The significance threshold was set at *p* < 0.05 for all analyses except for mediation analyses, where significance was determined if the bootstrapped mean coefficient (β) did not include the value 0.

## 3 Results

### 3.1 Baseline

The study included 4183 participants, comprising 2223 (53.1%) females and 1960 (46.9%) males. The mean (SD) age of the population was 51.8 (13.5) years. At baseline, MetS was identified in 1943 (46.4%) participants. The mean age (SD) of participants with MetS was 56.9 (11.7) years, compared to 47.4 (13.4) years for those without MetS (*p* < 0.001) (Table 1).

**TABLE 1:** Characteristics of the population according to presence of MetS at baseline.

	MetS		*p* ^a^
	No	Yes	
	Mean (SD)		
Age (years)	47.4 (13.4)	56.9 (11.7)	< 0.001
Education (years)	13.6 (3.9)	11.7 (4.0)	< 0.001
Systolic blood pressure (mmHg)	127.7 (17.4)	143.1 (18.4)	< 0.001
Diastolic blood pressure (mmHg)	75.8 (10.0)	82.9 (10.6)	< 0.001
Cholesterol (mmol/L)	5.2 (0.9)	5.4 (1.0)	< 0.001
HDL cholesterol, female (mmol/L)	1.7 (0.4)	1.4 (0.3)	< 0.001
HDL cholesterol, male (mmol/L)	1.4 (0.3)	1.2 (0.3)	< 0.001
Triglycerides (mmol/L)	1.1 (0.5)	1.8 (1.1)	< 0.001
Glucose (mmol/L)	5.6 (0.6)	6.2 (1.1)	< 0.001
BMI (kg/m^2^)	24.6 (3.3)	29.5 (4.0)	< 0.001
Waist circumference, female (cm)	81.0 (9.7)	95.6 (10.7)	< 0.001
Waist circumference, male (cm)	90.0 (8.0)	105 (8.6)	< 0.001
Number of MetS components	1.4 (0.9)	3.8 (0.8)	< 0.001

	*N* (%)		*p* ^b^
Sex (females)	1255 (57.3)	934 (48.1)	< 0.001
Current smokers	439 (20.0)	363 (18.7)	0.290
CRS categories			< 0.001
I	657 (30.0)	522 (26.9)	
II	1051 (48.0)	899 (46.3)	
III	482 (22.0)	522 (26.9)	

^a^ Welch's *t*‐test.

^b^ Chi‐squared test.

**FIGURE 1:**
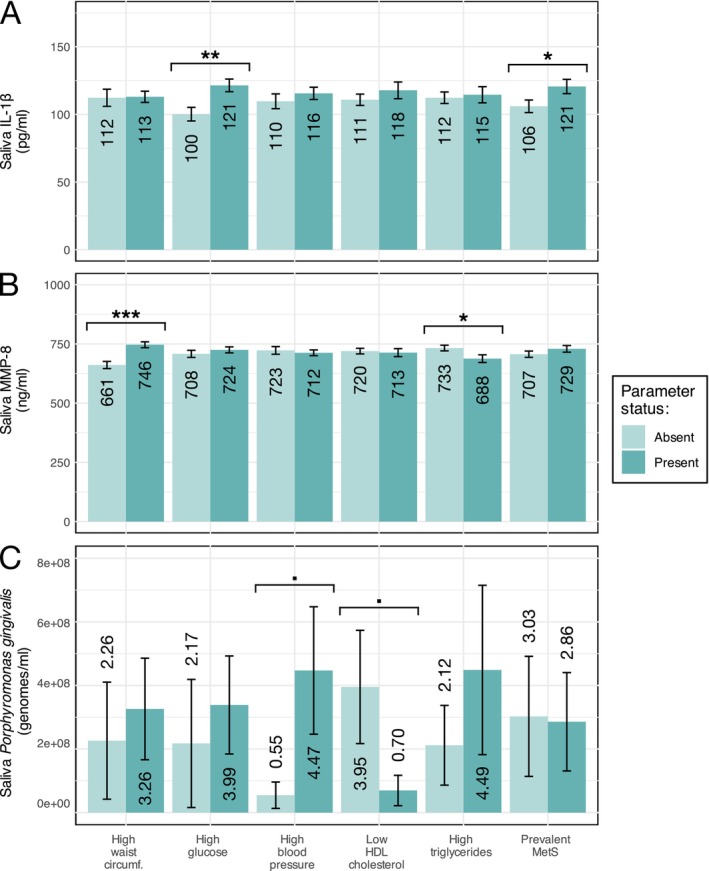
CRS biomarkers and positive MetS components. CRS biomarkers, saliva IL‐β (A), MMP‐8 (B) and *P. gingivalis* (C) were determined at baseline for 4183 participants to compose the CRS categories. Mean concentrations of each biomarker are shown as regards the presence of MetS components and prevalent MetS. The error bar depicts SE, and the mean value in each category is presented numerically. Statistical significance (Welch's *t*‐test) is presented symbolically above each variable in the following manner: •*p* < 0.1, *p < 0.05, **p < 0.01, ***p < 0.001.

The mean concentrations of CRS biomarkers with regard to MetS components and diagnoses at baseline are presented in Figure 1. Saliva IL‐1β concentrations were significantly higher in participants with high glucose levels (*p* < 0.01) and those with MetS (*p* < 0.05). Saliva MMP‐8 concentrations were higher in participants with high waist circumference (*p* < 0.001) and lower in those with high triglyceride concentrations (*p* < 0.05). Saliva *P. gingivalis* concentrations were higher in participants with high systolic blood pressure (*p* < 0.05) and lower in those with low HDL cholesterol levels (*p* < 0.05).

Of the participants in CRS I, CRS II and CRS III categories, metabolic syndrome was present in 522 (26.9%), 899 (46.3%) and 522 (26.9%) (*p* < 0.001), respectively (Table 1). Participants belonging to the CRS III group had a significantly higher prevalence of MetS compared to those with CRS I (*p* = 0.0004) and CRS II (*p* = 0.003), whereas there were no differences between low and moderate risk groups (*p* = 0.338).

**FIGURE 2:**
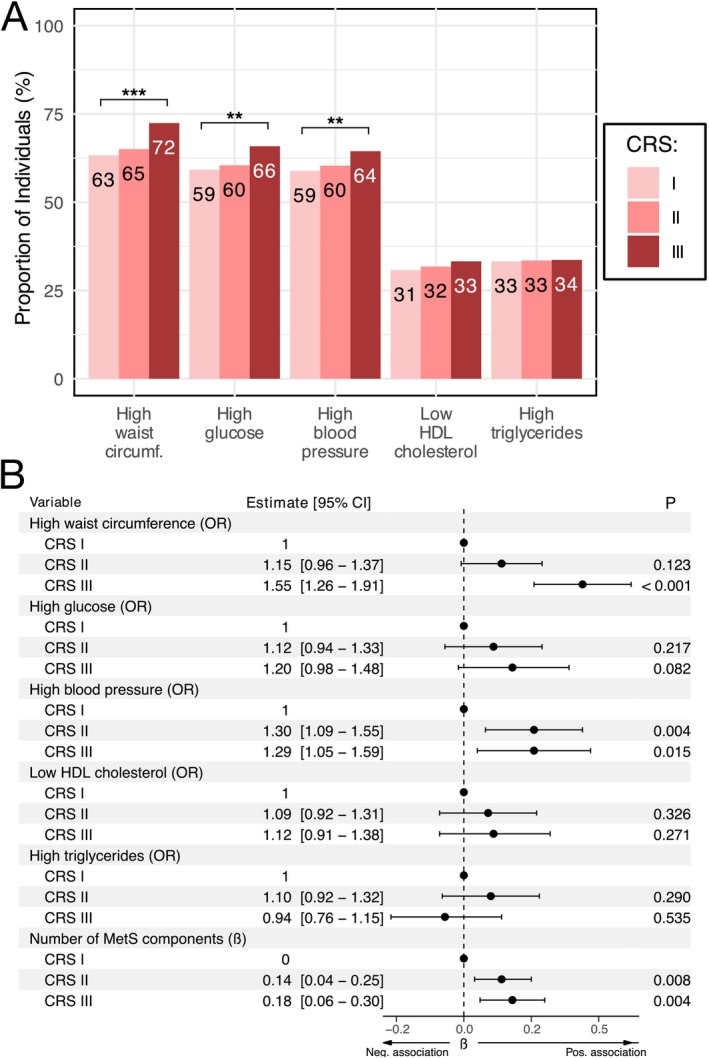
Association of CRS categories with positive MetS components at baseline. Presence of metabolic syndrome components were assessed at baseline for 4183 participants with CRS categories. (A) Proportion of individuals with positive MetS components in different CRS categories. (B) Association of CRS categories with positive MetS components in models adjusted for age, sex, smoking status, diet, physical activity, number of education years, household income and interaction between age and sex. Associations of CRS categories with MetS components are presented numerically in ORs and visually as log‐ORs, whereas the number of MetS components is presented as β‐estimates from a linear regression model. Statistical significance (Chi‐squared linear‐to‐linear test) is presented symbolically above each variable in panel A in the following manner: •*p* < 0.1, *p < 0.05, **p < 0.01, ***p < 0.001.

Higher CRS categories were associated with a higher prevalence of positive MetS components. The differences were statistically significant for high waist circumference (*p* < 0.001), elevated glucose levels (*p* < 0.01) and high blood pressure (*p* < 0.01) (Figure 2A). The mean number (SD) of positive MetS components increased with increasing CRS categories (*p* < 0.001), CRS I: 2.47 (1.48), CRS II: 2.52 (1.49) and CRS III: 2.70 (1.44). In multivariate analyses adjusted for age, sex, smoking, education years, diet, physical activity, household income and age–sex interaction (model 3), the number of MetS components, high waist circumference and high systolic blood pressure were associated significantly with increasing CRS groups, whereas no significant associations were observed for high levels of glucose, low HDL cholesterol or high triglycerides (Figure 2B).

**FIGURE 3:**
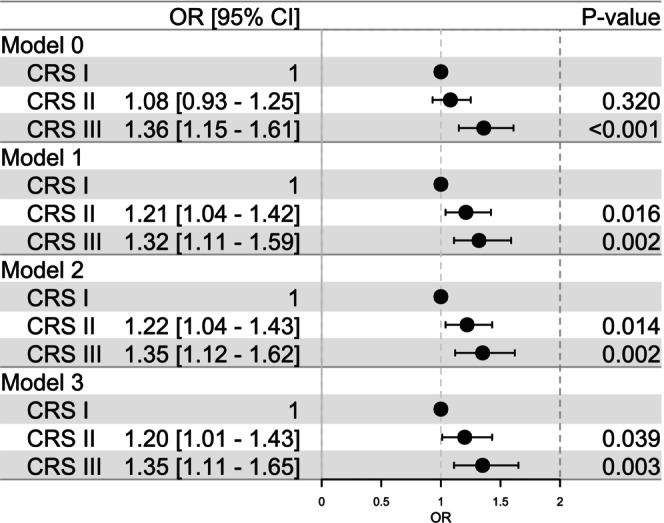
Association of CRS categories with prevalent MetS. Presence of metabolic syndrome was assessed at baseline in 4183 participants with CRS categories. The associations were analysed with binary logistic regression models with increasing adjustment: M0, crude unadjusted model; M1, adjusted for age and sex; M2, adjusted further for smoking status and number of education years; and M3, adjusted further for diet, physical activity, household income and interaction between age and sex. Odds ratio (OR) with 95% confidence intervals are shown.

In fully adjusted logistic regression models (model 3), both CRS II and CRS III were associated with higher odds (95% CI) of prevalent MetS compared with the CRS I category (CRS II OR: 1.20 [1.01–1.43], *p* = 0.039; CRS III OR: 1.35 [1.11–1.65], *p =* 0.003) (Figure 3). The interaction between age and sex was significant (OR 1.02 [1.01–1.03], *p* = 0.003), suggesting that age had a stronger effect on MetS risk in women than in men. In sensitivity analyses, the odds for prevalent MetS were highest in the age group 45–54 years, in women and in non‐smokers (Tables S1–S3).

### 3.2 Follow‐Up, Longitudinal

The follow‐up study included 1047 participants who underwent a clinical re‐examination and had available information. Of these participants, 429 (41.0%) had been diagnosed with MetS at baseline, while 618 had no prior MetS diagnosis. In the latter group, 128 (20.7%) developed MetS during the follow‐up period, while 490 (79.3%) remained MetS‐free. The participants with incident MetS were older (*p* < 0.001) and less educated (*p* = 0.013) than those who remained MetS‐free (Table 2).

**TABLE 2:** Baseline characteristics of subjects with incident MetS in the follow‐up.

	MetS		*p* ^a^
	No	Yes	
	Mean (SD)		
Age (years)	47.2 (13.1)	53.1 (12.5)	< 0.001
Education (years)	14.3 (3.9)	13.3 (4.2)	0.013
Systolic blood pressure (mmHg)	123.8 (15.4)	131.0 (18.0)	< 0.001
Diastolic blood pressure (mmHg)	74.5 (9.6)	79.0 (9.7)	< 0.001
Cholesterol (mmol/L)	5.1 (0.9)	5.3 (0.9)	0.009
HDL cholesterol, female (mmol/L)	1.8 (0.4)	1.6 (0.3)	< 0.001
HDL cholesterol, male (mmol/L)	1.4 (0.3)	1.3 (0.3)	0.302
Triglycerides (mmol/L)	1.0 (0.5)	1.1 (0.4)	0.024
Glucose (mmol/L)	5.6 (0.6)	5.7 (0.5)	0.223
BMI (kg/m^2^)	23.8 (2.9)	27.1 (3.3)	< 0.001
Waist circumference, female (cm)	78.4 (8.3)	89.1 (10.4)	< 0.001
Waist circumference, male (cm)	89.2 (7.9)	96.1 (7.2)	< 0.001
Number of MetS components	1.2 (0.9)	1.9 (0.8)	< 0.001

	*N* (%)		*p* ^b^
Sex (females)	292 (59.6)	79 (61.7)	0.662
Current smokers	87 (17.8)	26 (20.3)	0.525
CRS categories			0.105
I	141 (28.8)	27 (21.1)	
II	233 (47.6)	61 (47.7)	
III	116 (23.7)	40 (31.2)	

*Note*: Included only participants without MetS at baseline = 618.

^a^ Welch's *t*‐test.

^b^ Chi‐squared test.

The distribution across CRS groups was as follows: 168 (27.2%) in CRS I; 294 (47.6%) in CRS II; and 156 (25.2%) in CRS III. There was a significant difference in the proportions of participants with or without incident MetS between the CRS I and CRS III groups (*p* = 0.047). There were no differences between the CRS I and CRS II groups (*p* = 0.268) or between the CRS II and CRS III groups (*p* = 0.287) (Table 2).

**FIGURE 4:**
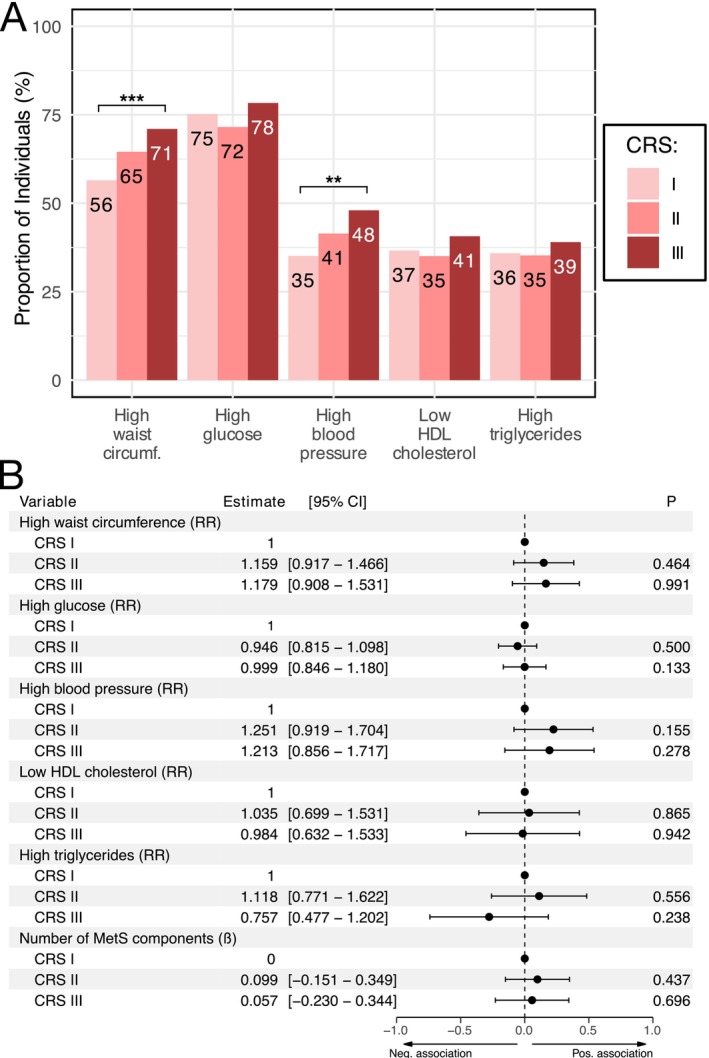
Association of CRS categories with positive MetS components in the follow‐up. Presence of metabolic syndrome components was assessed at follow‐up examination for 618 participants without prevalent MetS and with CRS categories. (A) Proportion of individuals with positive MetS components in different CRS categories. (B) Association of CRS categories with positive MetS components in models adjusted for age, sex, smoking status, number of education years, diet, physical activity, household income and interaction between age and sex. Associations of CRS categories with MetS components are presented numerically in RRs and visually as log‐RRs, whereas the number of MetS components is presented as β‐estimates from a linear regression model. Statistical significance (Chi‐squared linear‐to‐linear test) is presented symbolically above each variable in panel A in the following manner: •*p* < 0.1, *p < 0.05, **p < 0.01, ***p < 0.001.

Higher CRS displayed a greater prevalence of MetS components in the follow‐up examination. Statistical significance was seen for high waist circumference (*p* < 0.001) and high blood pressure (*p* < 0.01) (Figure 4A). However, fully adjusted modified Poisson regression models showed no significant associations between higher CRS categories and individual MetS components (Figure 4B).

**FIGURE 5:**
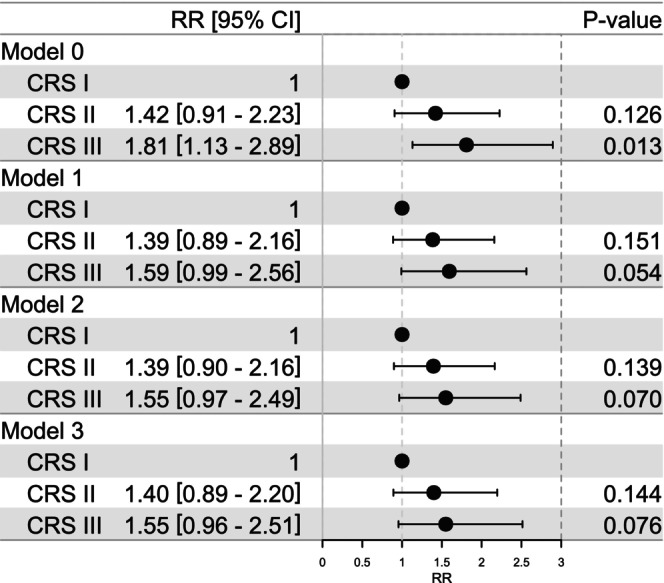
Association of CRS categories with incident MetS. Presence of metabolic syndrome was assessed during the follow‐up examination for 618 participants without MetS at baseline and with available CRS categories. The associations were analysed with modified Poisson regression models with increasing adjustment: M0, crude unadjusted model; M1, adjusted for age and sex; M2, adjusted further for smoking status and number of education years; and M3, adjusted further for diet, physical activity, household income and the interaction between age and sex. Risk ratios (RR) with 95% confidence intervals are shown.

CRS III was associated with a higher relative risk (RR) of MetS incidence in an unadjusted modified Poisson regression model with an RR of 1.81 (95% CI [1.13–2.89], *p* = 0.013) compared to CRS I. However, adjusting for covariates abolished the significance to *p* = 0.054–0.076. In a fully adjusted model, CRS III approached significance with an RR for incident MetS of 1.55 (95% CI [0.96–2.51], *p* = 0.076) (Figure 5). In sensitivity analyses, the RR was highest in the age group 35–44 years (3.10, 95% CI [0.90–10.7], *p* = 0.074), women (2.06, 95% CI [1.08–3.94], *p* = 0.028) and non‐smokers (1.78, 95% CI [1.01–3.14], *p* = 0.046) (Tables S1–S3). Mediation analyses identified higher inflammation (CRP), but not insulin resistance (HOMA‐IR), as a significant mediator between CRS and incident MetS (Table S4).

## 4 Discussion

In this population‐based study with a follow‐up re‐examination after 7 years, we found that the cumulative risk score for periodontitis (CRS) had an association with both prevalent and incident MetS. The association at baseline was independent of confounders. In prospective analyses, CRS was significantly associated with the risk of incident MetS only in subgroup analyses of women or non‐smokers. Systemic inflammation as measured by CRP concentrations mediated the risk of CRS with incident MetS.

When examining the associations between CRS and individual MetS components at baseline in our study, the most significant ones were for waist circumference, hyperglycaemia and hypertension as well as the number of MetS components. Our results align with and the estimates are on a similar range as in several previous findings (Ayuthaya et al. 2024; Campos et al. 2022; Saito et al. 2024; Sakurai et al. 2019) and the meta‐analyses of 38 studies, where obesity (OR 1.08), hyperglycaemia (OR 1.18), hypertension (OR 1.11) and a gradual increase of MetS components were associated with periodontitis (Campos et al. 2022). The meta‐analyses also identified a significant association between periodontitis and low HDL cholesterol (OR 1.16) but not with high triglyceride concentrations (Campos et al. 2022), whereas neither of the lipid levels was associated with periodontitis in the present study. These results are in conflict with two recent studies using metabolomics and a meta‐analysis, where periodontitis was associated with high triglyceride and low HDL cholesterol concentration (Basdorf et al. 2024; Ma et al. 2024; Salminen et al. 2024). The present analyses were not only based on the threshold levels of lipids but also considered users of lipid‐lowering medications as dyslipidemic participants, which should improve correct classifications of the individuals.

At baseline, in our study, CRS was associated with prevalent MetS with an OR of 1.35 (1.11–1.65), which is similar as in a recent meta‐analysis presenting an OR of 1.38 (1.26–1.51) (Daudt et al. 2018). Risk of MetS was also associated with increasing CRS categories, which aligns with a meta‐analysis suggesting a dose–response effect (Rosário‐Dos‐Santos et al. 2023). Among the five longitudinal studies, a 10‐year follow‐up study by Ayuthaya et al. found that each millimetre increase in mean clinical attachment loss (CAL) was associated with a 12% increase in the risk of developing MetS and that in severe periodontitis the RR for incident MetS was 1.43 (1.02–2.02) (Ayuthaya et al. 2024). An 8‐year follow‐up study by Saito and co‐workers reported an RR of 1.30 (1.01–1.67) for incident MetS in participants with ≥ 6 mm probing pocket depth (PPD) (Saito et al. 2024). Thus, the risk estimates were slightly lower compared to our study displaying an adjusted RR of 1.55 (0.96–2.51) in the whole population, 2.06 (1.08–3.94) in women and 1.78 (1.01–3.14) in non‐smokers. These two recent studies included 2161 and 4747 participants, respectively. Therefore, our prospective analysis including only 618 participants may have been underpowered. Three earlier studies concluded that the presence of pathological PPD was associated with an increased risk of developing a higher number of MetS components during the follow‐up (Morita et al. 2010; Saito et al. 2024; Sakurai et al. 2019). In our study, increasing CRS was correlated with the frequency of all developing MetS components. However, none of these reached statistical significance in the fully adjusted models. One earlier study found no association between developing MetS or its components and baseline periodontitis based on the community periodontal index of treatment needs (CPITN), but the follow‐up of 1 year might have been too short (Adachi and Kobayashi 2020).

Definition of periodontitis in our study was based on saliva biomarkers at baseline, whereas earlier studies investigating incident MetS have used different parameters. Three studies were based on CPITN measurements using either the presence of PPD ≥ 4 mm or ≥ 6 mm to define periodontitis or severe periodontitis (Morita et al. 2010; Saito et al. 2024; Sakurai et al. 2019). One study was based on CDC/AAP periodontal case definitions and dividing mean CAL and PPD measures into quintiles for severity (Ayuthaya et al. 2024). CRS was originally established in a case–control study including advanced periodontitis patients with at least 14 teeth with PPD ≥ 4 mm (Gürsoy et al. 2011) and later validated to distinguish healthy participants from patients with moderate to severe periodontitis (moderate to severe alveolar bone loss (ABL) and at least four sites with PPD of ≥ 4 mm) (Salminen et al. 2014). Thus, the CRS was later validated in a cohort where also radiographic data was available. The prevalence of CRS III (24.2%) in the present study was close to the frequency of ≥ 8 teeth with PPD of ≥ 4 mm (~23%) observed in a population‐based survey conducted in Finland in 2011 (Suominen et al. 2018).

One Mendelian randomisation (MR) study suggested that periodontitis may precede hypertension (Czesnikiewicz‐Guzik et al. 2019), whereas other studies have shown that it displays significant genetic correlations with several cardiometabolic traits, such as waist‐to‐hip ratio, BMI, obesity, stroke, type‐2 diabetes, hypertension and myocardial infarction (Salminen et al. 2025; Shungin et al. 2019). Another MR study, however, did not find a causal relationship of periodontitis with MetS (Yan et al. 2024). The relationship between oral diseases and metabolic disorders may begin already in early life because childhood oral infections are associated with adverse metabolic parameters and MetS in adulthood (Pussinen et al. 2020). Both diseases have a multifactorial aetiology, with multiple interactions of various genetic and environmental factors complicating the association. In addition to shared genetic susceptibility and unhealthy lifestyle, mechanisms combining the two diseases include systemic inflammation, altered/low diversity of the oral and gut microbiomes and oxidative stress (Pirih et al. 2021). Indeed, the mediation analyses of the present work identified systemic inflammation as a significant mediator between baseline CRS and incident MetS. However, further research is needed to determine whether periodontitis is a modifiable risk factor for MetS.

The strengths of our study include its population‐based design and follow‐up examinations to determine the incidence of MetS. However, in the cross‐sectional analyses, reverse causality cannot be ruled out, since MetS and periodontitis share similar risk factors and are both linked to systemic inflammation (Campos et al. 2022). Although the main MetS risk factors were considered in our analyses, residual confounding due to unmeasured confounders may be present (McCracken et al. 2018; Pirih et al. 2021). The main limitation was the restricted number of participants in the follow‐up, while only 618 individuals were MetS‐free at baseline. The risk of having periodontitis was determined using validated biomarkers and CRS, which cannot be considered a limitation but rather an innovation. Until now, CRS has been used to investigate its association with obesity (Syrjäläinen et al. 2019) and dietary inflammatory index (Syrjäläinen et al. 2024), whereas other studies have concentrated on validating the biomarker score (Salminen et al. 2014; Gürsoy et al. 2018; Liukkonen et al. 2018, 2020) after the initial discovery case–control study (Gürsoy et al. 2011). As with other salivary biomarkers, CRS may be biased in participants with advanced tooth loss or in edentulous participants (Palm et al. 2014). During the time when the present cohort was collected, ≤ 10% of Finnish adults were edentulous, and teeth were typically lost in subjects of ≥ 65 years of age (https://www.julkari.fi/). In the present study, the proportion of ≥ 65‐year‐olds was 32%; however, we did not have any information about their oral health parameters or periodontal treatment history. Despite the limitations, salivary biomarkers would provide a valuable tool in periodontal research. Non‐invasively and easily collectable sample material and biomarkers, which can be determined in a routine laboratory setting, would be cost effective and enable investigating large‐scale studies that are required to use modern techniques such as omics tools. Thus, the performance of the existing biomarkers and their combinations should be carefully examined both in periodontal diseases and in periodontitis‐systemic conditions.

## 5 Conclusion

Our population‐based study using saliva biomarkers to define periodontitis confirmed that periodontitis is associated with an increased risk of prevalent MetS, especially the number of MetS components, abdominal obesity, hyperglycaemia and hypertension. The results also suggest that periodontitis may have a positive trend with increased risk of incident MetS, especially in two groups of participants devoid of established cardiovascular risk factors, that is, women and non‐smokers.

## Author Contributions

Conceptualization: Jenni Kinnunen, Satu Männistö, Veikko Salomaa, Pekka Jousilahti, Eija Könönen, Ulvi Kahraman Gürsoy, Aino Salminen, Pirkko Pussinen. Data curation: Jenni Kinnunen, Kari Koponen, Oleg Kambur, Aki S. Havulinna. Laboratory work: Muhammed Manzoor, Katariina Aarnisalo, Verneri Nissilä, Ulvi Kahraman Gürsoy, Pirkko Pussinen. Formal analysis: Jenni Kinnunen, Kari Koponen, Oleg Kambur. Project administration: Pirkko J. Pussinen, Satu Männistö, Eija Könönen. Visualisation: Jenni Kinnunen, Kari Koponen. Writing – original draft: Jenni Kinnunen, Pirkko Pussinen. Writing – review and editing: Kari Koponen, Oleg Kambur, Muhammed Manzoor, Katariina Aarnisalo, Verneri Nissilä, Satu Männistö, Veikko Salomaa, Pekka Jousilahti, Eija Könönen, Ulvi Kahraman Gürsoy, Aki S. Havulinna, Aino Salminen.

## Conflicts of Interest

The authors declare no conflicts of interest.

## Supporting information


**Data S1:** jcpe70042‐sup‐0001‐supinfo.pdf.

**Data S2:** jcpe70042‐sup‐0002‐supinfo.docx.

## Data Availability

The data that support the findings of this study are available from the corresponding author upon reasonable request.
