# Supplementary material for: The Association of Periodontitis With Risk of Prevalent and Incident Metabolic Syndrome
Source: J Clin Periodontol. 2025 Sep 23;53(1):107–16. doi: 10.1111/jcpe.70042 (PMC12695448; doi:10.1111/jcpe.70042)
Supplement: Supplementary file 1 — Data S1: jcpe70042‐sup‐0001‐supinfo.pdf. [file JCPE-53-107-s001.pdf]

Joint Municipal Board for the Hospital District of Helsinki and Uusimaa
Ethics Committee for Epidemiology and Public Health

Opinion of the Ethics Committee for Epidemiology and Public Health 87/2001

Ref. 558/E3/2001
As the Committee Chair disqualified himself from the discussion concerning the research plan and as the Vice-chair could not attend the meeting, the committee members chose among themselves Mikko Nissinen as chair.

Research plan identifier / name
National FINRISK 2002 Study

Commissioning party / Financier
National Public Health Institute (KTL), ETEO
Lappi and Länsi-Pohja hospital districts, Matti Kakko,PO Box 8041, 96101 Rovaniemi
University of Oulu, Simo Näynä, PO Box 5000, Oulu

Person in charge of the research
Aulikki Nissinen, Research Professor, D.Med.Sc., KTL, ETEO

Contact person for the research
Tiina Laatikainen, Senior Researcher, D.Med.Sc., KTL, ETEO

Researchers / Research institutes
Erkki Vartiainen, Research Professor, KTL
Pekka Jousilahti, Adjunct Professor, KTL
Tiina Laatikainen, D.Med.Sc., KTL
Jouko Sundvall, M.Sc., KTL
Antti Uutela, Adjunct Professor
Georg Alfthan, D.Phil., KTL
Pirjo Pietinen, Research Professor, KTL
Liisa Valsta, D.Med.Sc, KTL
Veikko Salomaa, Adjunct Professor, KTL
Jussi Huttunen, Director General, KTL

Documents submitted to the Ethics Committee
Covering letter by the responsible researcher, 7 Dec 2001
Form for requesting opinion 5B, 7 Dec 2001
Research plan, 5 Dec 2001
Written consent document (information for study subjects + written consent form), 5 Dec 2001

The Ethics Committee for Epidemiology and Public Health of the Joint Municipal Board for the Hospital District of Helsinki and Uusimaa has discussed the above documents in its meetings on 19 December 2001.

Opinion
Final hearing. The Committee gives a favourable opinion to the research plan.
The written consent document complies with the regulations laid down in Acts 523/1999 and 488/1999 and in Decree 986/1999. The Committee proposes, however, that the researchers amend paragraph 5 of the information to study subjects so that it complies with what has been said in the research plan concerning the analysing of DNA samples.
The final written consent document (information to study subjects + written consent form) should be sent in one copy without the form 5B to the Committee Secretary for information and archiving by 12 a.m. on 21 January 2002.

June 20, 2006

FINRISK 2007 (229/E0/06)

229/E0/2006

TMKE10 Section 157

Presenter: Professor Leena Kivisaari

A new clinical medical study has been submitted to the Committee for consideration.

The study is a population study whose purpose is to monitor the level of and changes in risk factors associated with cardiovascular diseases and certain other public health challenges in the middle-aged population in Finland.

Person in charge of the study

Erkki Vartiainen, MD, Research Professor

Party commissioning / funding the study

National Public Health Institute of Finland

List of appended application documents

Research plan, versio1_02062006
Summary of the study in Finnish, versio1_02062006
Notification to subjects in Finnish, versio1_02062006
Notification to subjects in Swedish, versio1_02062006
Consent form for subjects in Finnish, versio1_02062006
Consent form for subjects in Swedish, versio1_02062006
Specimen case report form (CRF), versio1_02062006
Assessment by the person in charge of the study concerning ethical compliance, versio1_02062006
Curriculum Vitae, versio1_02062006
Summary of research funding, versio1_02062006
Personal data file description, versio1_02062006
Covering letters to subjects, versio1_02062006
Application (form), June 5, 2006
List of appendices, 02062006

Proposal for decision

The Ethics Committee to decide:
1. that the study be approved in the meeting procedure,
2. that the processing fee be set at EUR 0 (Decree of the Ministry of Social Affairs and Health 1396/2004, section 1(3)).

Processing

Docent Markus Perola and Docent Jarmo Virtamo recused themselves from discussion of the matter and left the meeting.

Decision

The Committee considers that the research plan and its appendices comply with the provisions of the Medical Research Act (488/99 and 295/04) and the Medical Research Decree (986/99 and 316/04), with the provisions on data protection and with the international ethical guidelines

concerning medical research and the status of research patients which pertain to research involving human subjects.

The Ethics Committee considers that the research plan is ethically acceptable and hereby decides

1. to issue a favorable statement regarding the study, provided that the following corrections are made in the documentation:
   - In the Consent Form, the paragraph concerning the subject's consent to the obtaining of information on his/her health from registers kept by various authorities and by units of health care, such as Statistics Finland, the National Research and Development Centre for Welfare and Health (STAKES), the Finnish Centre for Pensions and the Social Insurance Institution of Finland (KELA) must be removed.

2. to charge a processing fee as proposed in the presentation.

Submitting of corrections/reports

The corrections made must be clearly indicated in the amended documents. Additions must be marked in bold italic text, and deletions by overstriking or with a gray highlight. The Committee discusses several projects at each meeting, and the volume of documents and appendices is massive. The experts cannot be expected to make a word-by-word comparison between the original and the corrected documents.

Please also append a copy of the extract from the minutes requiring the corrections or reports submitted.

Please submit the corrected documents to the central registry of the Hospital District of Helsinki and Uusimaa, PO Box 100, 00029 HUS, Finland.

Further information

Presenter Leena Kivisaari, tel. +358 50 411 291
Committee Secretary Heli Ådjers, tel. +358 9 471 71257

Extract from non-verified minutes certified correct
June 27, 2006

(signature)
Heli Ådjers
secretary

Sent for information   June 27, 2006

Coordinating Ethics Committee   Section 157   June 20, 2006

DISTRIBUTION   Vartiainen Erkki
Peltonen Markku

Coordinating Ethics Committee | Section 46 | February 20, 2007

229/E0/06 AMENDMENT OF RESEARCH PLAN

229/E0/2006

TMKE10 Section 46

Presenter: Docent Riitta-Sisko Koskela

Title of study: FINRISK 2007

Description: The research plan was approved at the meeting of the Ethics Committee on June 20, 2006, and a favorable statement was issued on it. A notification of an amendment to the research plan has been submitted to the committee for consideration.

The study is a population study whose purpose is to monitor the level of and changes in risk factors associated with cardiovascular diseases and certain other public health challenges in the middle-aged population in Finland.

Person in charge of the study
Erkki Vartiainen, MD, Research Professor

Party commissioning the study
Investigator-driven research

Party funding the study
National Public Health Institute of Finland

List of documents appended to the application

Appendices to the application (list), dated January 11, 2007, versio3/150107
Research plan, versio3/150107
Summary of the study in Finnish, versio2/301006
Notification to subjects in Finnish, dated January 15, 2007, versio3/150107
Consent form for subjects in Finnish, versio3/150107
Covering letters to subjects in Finnish, versio3/150107
Stool sample: Subject's form and instructions for taking sample, versio1/150107

Proposal for decision: The Ethics Committee to decide

1. that the study be approved in the meeting procedure,

2. that the processing fee be set at EUR 0 (Decree of the Ministry of Social Affairs and Health 1390/2006, section 1(3)).

Processing: Professor Jarmo Virtamo recused himself from discussion of the matter and left the meeting. Discussion of the matter was chaired by Docent Riitta-Sisko Koskela.

Decision	The Ethics Committee hereby decides<br><br>1. to approve the amendment to the research plan,<br><br>2. to charge a processing fee as proposed in the presentation.
Further information	Presenter Riitta-Sisko Koskela, tel. +358 50 531 3496<br>Committee Secretary Heli Ådjers, tel. +358 9 471 71257,<br>+358 50 427 9123
	Extract from non-verified minutes certified correct<br><br>(signature)<br>Heli Ådjers<br>secretary
Sent for information	February 27, 2007
DISTRIBUTION	Vartiainen Erkki<br>Peltonen Markku

Koordinoiva eettinen toimikunta § 90 03.04.2007

229/E0/06 MUUTOS TUTKIMUSSUUNNITELMAAN

229/E0/2006

TMKE10 § 90

Esittelijä: Dosentti Riitta-Sisko Koskela

Julkisuus: Salassa pidettävä (JulkL 621/1999, 24 § mom. kohta)

Tutkimuksen nimi: FINRISKI 2007

Kuvaus: Tutkimussuunnitelma on hyväksytty eettisen toimikunnan kokouksessa 20.6.2006 ja siitä on annettu puoltava lausunto. Toimikunnan käsiteltäväksi on toimitettu ilmoitus tutkimussuunnitelman muutoksesta.

Kyseessä on väestötutkimus, jonka tarkoituksena on seurata sydän- ja verisuonitautien sekä eräiden muiden kansantautien riskitekijöiden tasoa ja muutosta keski-ikäisessä väestössä Suomessa.

Tutkimuksesta vastaava henkilö: LT, tutkimusprofessori Erkki Vartiainen

Tutkimuksen toimeksiantaja: Tutkijalähtöinen tutkimus

Tutkimuksen rahoittaja: Kansanterveyslaitos

Toimitetut asiakirjat:
Saate-/selvityskirje, 18.3.2007
Kutsukirje, muille paitsi ulostenäyte-alaotokseen kuuluvat
Kutsukirje, ulostenäyte-alaotokseen kuuluvat
Tutkittavan tiedote, muille paitsi ulostenäyte-alaotokseen kuuluvat
Tutkittavan tiedote, ulostenäyte-alaotokseen kuuluvat
Suostumusasiakirja, muille paitsi ulostenäyte-alaotokseen kuuluvat
Suostumusasiakirja, ulostenäyte-alaotokseen kuuluvat
Sokeri- ja rasva-aineenvaihduntatutkimus -kyselylomake
Ruoankäyttökysely
Hyvinvointikysely ravinnosta ja liikunnasta
Kyselylomake tupakoinnista
Ulostenäytteen keräyslomake
Ulostenäytteen keräysohjeet
Tutkimussuunnitelman hyväksyvä lausuntokopio, 20.6.2006

Päätösesitys: Eettinen toimikunta päättää

1. tutkimussuunnitelman muutoksen hyväksymisestä kokouskäsittelyssä,

2. periä lausuntomaksuna 0 euroa (STM:n asetus 1390/2006, 1 § 3 mom.).

Asian käsittely: Professori Jarmo Virtamo poistui esteellisenä asian käsittelyn ajaksi. Puheenjohtajana tämän asian käsittelyn ajan toimi dosentti Riitta-Sisko Koskela.

Päätös

Eettinen toimikunta päättää

1. hyväksyä tutkimussuunnitelman muutoksen,

2. periä käsittelymaksun esityksen mukaisesti.

Lisätietoja

Esittelijä Riitta-Sisko Koskela p. 050-531 3496
Toimikuntasihteeri Heli Ådjers p. (09) 471 71257, 050 427 9123

Otteen tarkastamattomasta pöytäkirjasta oikeaksi todistaa 12.4.2007

Heli Ådjers
sihteeri

Lähetetty tiedoksi 12.4.2007

JAKELU

Vartiainen Erkki
Peltonen Markku

332/13/03/00/13 UUSI TUTKIMUSSUUNNITELMA, 2. KÄSITTELY

332/13/03/00/2013

TMK00 § 6

Esittelijä

Ylilääkäri Juhani Partanen

Julkisuus

Salassa pidettävä (JulkL 621/1999, 24 §)

Tutkimuksen nimi

DILGOM 2014- SEURANTATUTKIMUS: THE EFFECT OF DIETARY, LIFESTYLE AND GENETIC FACTORS ON THE DEVELOPMENT OF OBESITY AND METABOLIC SYNDROME/ RAVINNON, ELINTAPOJEN JA PERINTÖTEKIJOIDEN VAIKUTUS LIHAVUUDEN JA METABOLISEN OIREYHTYMÄN SYNTYYN

Kuvaus

Koordinoiva eettinen toimikunta käsitteli kokouksessaan 17.12.2013 uuden tutkimussuunnitelman ja päätti jättää sen pöydälle lisäselvityksiä ja korjauksia varten. Nyt toimikunnan käsiteltäväksi on toimitettu pyydetyt korjaukset.

Kyseessä on kliininen lääketieteellinen tutkimus, jonka tavoitteena on tuottaa uutta tietoa viidellä lihavuuden ja metabolisen syndrooman syntyyn liittyvältä osa-alueelta: 1) elintavat (ravinto, liikunta, tupakointi, alkoholinkäyttö, uni), 2) psykososiaaliset tekijät, 3) aineenvaihdunta ja hormonaaliset tekijät, 4) altistuminen ympäristön kemikaalijäämille, sekä 5) perintötekijät.

Tutkimuksesta vastaava henkilö

Tutkimusprofessori, yleislääketieteen ja terveydenhuollon erikoislääkäri, FT Pekka Jousilahti, Terveyden ja hyvinvoinnin laitos, Helsinki

Tutkimuspaikat

Terveyden ja hyvinvoinnin laitos ja Helsingin yliopisto

Tutkimuksen toimeksiantaja

Tutkijalähtöinen tutkimus.

Tutkimuksen rahoitus

THL (50%), HY (20%), Folkhälsan (10%), Vainion säätiö (10%) ja Sydäntutkimussäätiö (10%)

Toimitetut asiakirjat

Saatekirje, Pekka Jousilahti 20.12.2013
Tutkimussuunnitelma, versio 2, 27.12.2013
Kutsu terveystutkimukseen, versio 2, 27.12.2013
Tiedote tutkittavalle, 27.12.2013 (HUS -leima)
Aikaisempi lausunto § 233, 17.12.2013
Saatekirje 30.12.2013 ja TVH:n oma arvio tutkimuksen eettisyydestä, versio 1, 21.11.2013
Saapumisilmoitus, TUKIJA 9.1.2014

Päätösesitys

Eettinen toimikunta päättää

1. asiasta kokouskäsittelyssä,

2. periä lausuntomaksuna 0 euroa (STM:n asetus 650/2013, 1 § 3 mom.).

Asian käsittely

Tutkimusprofessori Markus Perola poistui kokouksesta esteellisenä asian käsittelyn ajaksi.

Päätös

Toimikunta katsoo, että tutkimussuunnitelma ja sen liiteasiakirjat noudattavat lääketieteellisestä tutkimuksesta annetun lain (488/99 myöh. muutoksineen) ja asetuksen (986/99 myöh. muutoksineen) säännöksiä, tietosuojasäännöksiä sekä niitä lääketieteellistä tutkimusta ja tutkimuspotilaiden asemaa koskevia kansainvälisiä velvoitteita, joita ihmiseen kohdistuvalta lääketieteelliseltä tutkimukselta edellytetään.

Eettinen toimikunta pitää tutkimussuunnitelmaa eettisesti hyväksyttävänä ja päättää

1. antaa siitä puoltavan lausunnon. Jos näytteitä aiotaan luovuttaa myöhemmin Yhdysvaltain liittovaltion (esim. NIH:n) biobankkiin kuten suunnitemassa todetaan, tulee suomalaisenkin potilasinformaation ja suostumuksen vastata sitä, mitä NIH näytteiden luovuttamisen yhteydessä nykyään edellyttää kun genomitietoa tallennetaan. Toimikunta ehdottaa, että tutkijat keskustelevat asiasta THL:n FUSION tutkijan kanssa, joilla on asiasta kokemusta.

Päätöstä koskeviin asiasisällöllisiin kysymyksiin vastaa tarvittaessa esittelijä.

2. periä lausuntomaksun esityksen mukaisesti.

Eettisen toimikunnan antamasta lausunnosta ei voi valittaa. Jos eettisen toimikunnan lausunto on kielteinen, toimeksiantaja voi saattaa asian uudelleen eettisen toimikunnan käsiteltäväksi. Alueellisen eettisen toimikunnan on toimeksiantajan pyynnöstä hankittava asiasta ennen uuden lausuntonsa antamista valtakunnallisen lääketieteellisen tutkimuseettisen toimikunnan lausunto (Tutkimuslaki 488/1999 myöh. muutoksineen).

Maksuvelvollinen, joka katsoo, että maksun määräämisessä on tapahtunut virhe, voi vaatia oikaisua. Oikaisuvaatimusohje on liitteenä.

Lisätietoja

Esittelijä Juhani Partanen, p. 09 471 73901, 040 717 6014
Puheenjohtaja Katja Käyhkö, p. 050 339 2688

Otteen tarkastamattomasta pöytäkirjasta oikeaksi todistaa

Helsingissä 21.1.2014

Terhi Ruuth
sihteeri

Lähetetty tiedoksi 21.1.2014

Liite Oikaisuvaatimusohje

Jakelu Tutkimusprofessori Pekka Jousilahti ja tutkija Annika Wennerström, Terveyden ja hyvinvoinnin laitos, PL 30, 00271 Helsinki

Koordinoiva eettinen toimikunta

Aika	14.01.2014 klo 15:30 - 18:05
Paikka	Biomedicum Helsinki 2 C, 3. krs., kliinisen farmakologian kokoushuone

Osallistujat

	Nimi	Tehtävä	Klo	Lisätiedot
LÄSNÄ	Käyhkö Katia	jäsen (pj)	15:30 - 18:05	Puheenjohtaja
	Koskela Riitta-Sisko	jäsen (vpj)	15:30 - 18:05	
	Lehtonen Lasse	jäsen (vpj)	15:30 - 18:05	
	Perola Markus	jäsen (vpj)	15:30 - 18:05	Esteellinen §:n 6 ajan
	Autti Taina	jäsen	15:30 - 18:05	
	Henriksson Lea	jäsen	15:30 - 18:05	
	Junttila Kristiina	jäsen	15:30 - 18:05	
	Kanerva Jukka	jäsen	15:30 - 18:05	
	Lumio Jukka	jäsen	15:30 - 18:05	
	Niemi Mikko	jäsen	17:00 - 18:05	Läsnä §:stä 5 lähtien
	Partanen Juhani	jäsen	15:30 - 17:15	Poistui §:n 7 aikana
	Vehmas Tapio	jäsen	15:30 - 18:05	
	Salenius Kaija	maall.jäsen	15:30 - 18:05	
	Ylikarjula Simo	maall.jäsen	15:30 - 18:05	
POISSA	Vilkman Sirkku	jäsen		
	Alttomäki Kristiina	varajäsen		
	Arola Matti	varajäsen		
	Gylling Helena	varajäsen		
	Karhu-Hämäläinen Anita	varajäsen		
	Lehikoinen Tuula	varajäsen		
	Notkola Veijo	maall.varajäsen		
	Puolakkainen Mirja	varajäsen		
	Suvisaari Jaana	varajäsen		
	Verkasalo Matti	varajäsen		
	Seppänen Laura	varajäsen		
	Ruuska Minna	sihteeri		
MUU	Ruuth Terhi	sihteeri	15:30 - 18:05	Sihteeri

Vastaanottaja Professori Markus Perola, THL, BM 1, PL 140, 00215 Helsinki

Tutkimuksen nimi
Mikrobiomi ja metabolomiikka tutkimus

Viite HUS/2158/2017

HUS Kesälomakauden eettinen toimikunta käsitteli tutkimussuunnitelmanne kokouksessaan 19.7.2017 § 1 ja pyysi siihen korjauksia. Toimikunnan valtuuttamana puheenjohtajalla on valtuudet hyväksyä toimikuntaan saapuneet pyydetyt korjaukset ja lisäselvitykset.

Tutkimussuunnitelman pyydetyt korjaukset ja lisäselvitykset käyvät ilmi toimitetuista asiakirjoista:

- Lausuntokopio § 1, 19.7.2017
- Kutsu, tiedote ja suostumus, 19.6.2017
- Tieteellisen tutkimuksen rekisteriseloste, 19.6.2017

Tutkimussuunnitelma ja sen liitteet täyttävät tutkimuslain (488/1999) 17 §:n 3 momentin mukaiset edellytykset.

Päätös

Toimikunnan puolesta puheenjohtaja päätti hyväksyä pyydetyt korjaukset/ lisäselvitykset ja antaa niistä puoltavan lausunnon.

Toimikunnan pyytämät korjaukset, lausunto on maksuton (STM:n asetus 1168/2014, 1 § 3 mom).

Vakuudeksi 23.8.2017

Helsingissä

Katia Käyhkö
Puheenjohtaja
Kesälomakauden eettinen toimikunta
