# Supplementary material for: The Association of Periodontitis With Risk of Prevalent and Incident Metabolic Syndrome
Source: J Clin Periodontol. 2025 Sep 23;53(1):107–16. doi: 10.1111/jcpe.70042 (PMC12695448; doi:10.1111/jcpe.70042)
Supplement: Supplementary file 2 — Data S2: jcpe70042‐sup‐0002‐supinfo.docx. [file JCPE-53-107-s002.docx]

**Supplementary material**

**The association of periodontitis with risk of prevalent and incident metabolic syndrome**

Jenni Kinnunen^1^, Kari Koponen^1, 2, 3^, Oleg Kambur^1, 2^, Muhammed Manzoor^4,^ Katariina Aarnisalo^4^, Verneri Nissilä^5,^ Satu Männistö^2^, Veikko Salomaa^2^, Pekka Jousilahti^2^, Eija Könönen^5,^

Ulvi Kahraman Gürsoy^5^, Aki S. Havulinna^2,6,7^, Aino Salminen^4^, Pirkko Pussinen^1, 4^

^1^ School of Medicine, Institute of Dentistry, University of Eastern Finland, 70211 Kuopio, Finland

^2^ Department of Public Health, Finnish Institute for Health and Welfare, 00271 Helsinki, Finland

^3^ Department of Bacteriology and Immunology, University of Helsinki, 00014 Helsinki, Finland

^4^ Oral and Maxillofacial Diseases, University of Helsinki and Helsinki University Hospital, 00014 Helsinki, Finland

^5^ Periodontology, Institute of Dentistry, University of Turku, 20520 Turku, Finland

^6^Institute for Molecular Medicine Finland, FIMM-HiLIFE, 00014 University of Helsinki, Finland

^7^Department of Computing, University of Turku, 20014 University of Turku, Finland

## **Supplementary Methods**

## ***Ethical Issues***

The DILGOM study complies with the Declaration of Helsinki, and it was approved by the Ethical Committee of the Hospital District of Helsinki and Uusimaa (decision numbers 229/E0/2006 and 332/13/03/00/2013). Informed and written consent was obtained from all participants.

## ***Study Population***

The National FINRISK studies are conducted every five years to monitor the health of the adult population in Finland (target age group 25-74 years). FINRISK 2007 is a population-based, random sample of 10,000 Finns (n=6258, participation rate 63%) (Borodulin et al., 2018). DIetary, Lifestyle and Genetic determinants of Obesity and Metabolic syndrome (DILGOM) is an expansion of FINRISK 2007 where all participants were invited to an extensive clinical examination (n=5024, participation rate of 80%). The recruitment protocol and details have been reported earlier (Kanerva et al., 2018). The clinical examinations were performed between April and June 2007.

Eligible participants of the baseline DILGOM with complete data (n=4581) were invited to take part in the DILGOM 2014 follow-up phase conducted between April and June 2014. Participation rate was 82% (n=3725). The study was divided into two groups: 1) 2423 (65%) did a self-assessment at home by using a measuring tape, which was mailed to them. This group was not included in the present follow-up. 2) 1312 (35%) participants living in the capital area underwent a similar clinical examination as conducted during the baseline phase and filled in a health-related questionnaire. The included clinical laboratory investigations enabled setting the MetS diagnosis. These participants comprised the follow-up group of the present study. A flow chart clarifying the study design is shown in **Figure S1**.

**References**

Borodulin, K., Tolonen, H., Jousilahti, P., Jula, A., Juolevi, A., Koskinen, S., Kuulasmaa, K., Laatikainen, T., Männistö, S., Peltonen, M., Perola, M., Puska, P., Salomaa, V., Sundvall, J., Virtanen, S. M., & Vartiainen, E. (2018). Cohort Profile: The National FINRISK Study. *International Journal of Epidemiology*, *47*(3), 696–696i. https://doi.org/10.1093/ije/dyx239

Kanerva, N., Harald, K., Männistö, S., Kaartinen, N. E., Maukonen, M., Haukkala, A., & Jousilahti, P. (2018). Adherence to the healthy Nordic diet is associated with weight change during 7 years of follow-up. *British Journal of Nutrition*, *120*(1), 101–110. https://doi.org/10.1017/S0007114518001344

**Table S1.** Sensitivity analyses for age groups in prevalent MetS**.**

|  |  |  |  |  |  |
| --- | --- | --- | --- | --- | --- |
| Age group | **Cases / total n** | **Model** |  | **OR (95% CI)** | **p-value** |
| 25-34 years | 89 / 530 | M0 | CRS II | 1.327 (0.770-2.356) | 0.319 |
|  |  |  | CRS III | 1.320 (0.665-2.607) | 0.423 |
|  |  | M1 | CRS II | 1.343 (0.775-2.396) | 0.303 |
|  |  |  | CRS III | 1.296 (0.649-2.576) | 0.459 |
|  |  | M2 | CRS II | 1.479 (0.845-2.669) | 0.180 |
|  |  |  | CRS III | 1.421 (0.703-2.866) | 0.324 |
|  |  | M3 | CRS II | 1.392 (0.788-2.529) | 0.264 |
|  |  |  | CRS III | 1.426 (0.703-2.887) | 0.322 |
| 35-44 years | 218 / 689 | M0 | CRS II | 1.350 (0.916-2.007) | 0.134 |
|  |  |  | CRS III | 1.241 (0.776-1.985) | 0.367 |
|  |  | M1 | CRS II | 1.374 (0.929-2.048) | 0.115 |
|  |  |  | CRS III | 1.205 (0.751-1.933) | 0.439 |
|  |  | M2 | CRS II | 1.345 (0.904-2.019) | 0.148 |
|  |  |  | CRS III | 1.163 (0.715-1.890) | 0.542 |
|  |  | M3 | CRS II | 1.334 (0.886-2.026) | 0.171 |
|  |  |  | CRS III | 1.070 (0.645-1.769) | 0.792 |
| 45-54 years | 353 / 859 | M0 | CRS II | 1.348 (0.966-1.890) | 0.081 |
|  |  |  | CRS III | 1.460 (0.989-2.160) | 0.057 |
|  |  | M1 | CRS II | 1.392 (0.994-1.959) | 0.056 |
|  |  |  | CRS III | 1.464 (0.990-2.174) | 0.058 |
|  |  | M2 | CRS II | 1.398 (0.994-1.973) | 0.055 |
|  |  |  | CRS III | 1.493 (1.004-2.226) | **0.048** |
|  |  | M3 | CRS II | 1.461 (1.029-2.087) | **0.035** |
|  |  |  | CRS III | 1.586 (1.053-2.398) | **0.028** |
| 55-64 years | 497 / 846 | M0 | CRS II | 1.182 (0.853-1.638) | 0.315 |
|  |  |  | CRS III | 1.425 (0.969-2.100) | 0.072 |
|  |  | M1 | CRS II | 1.191 (0.858-1.653) | 0.296 |
|  |  |  | CRS III | 1.406 (0.955-2.077) | 0.085 |
|  |  | M2 | CRS II | 1.168 (0.838-1.626) | 0.359 |
|  |  |  | CRS III | 1.371 (0.928-2.032) | 0.114 |
|  |  | M3 | CRS II | 1.171 (0.830-1.651) | 0.369 |
|  |  |  | CRS III | 1.353 (0.905-2.029) | 0.141 |
| 65-74 years | 541 / 805 | M0 | CRS II | 0.853 (0.592-1.223) | 0.389 |
|  |  |  | CRS III | 1.231 (0.829-1.827) | 0.303 |
|  |  | M1 | CRS II | 0.859 (0.596-1.233) | 0.411 |
|  |  |  | CRS III | 1.246 (0.838-1.854) | 0.277 |
|  |  | M2 | CRS II | 0.865 (0.597-1.248) | 0.441 |
|  |  |  | CRS III | 1.281 (0.853-1.926) | 0.233 |
|  |  | M3 | CRS II | 0.870 (0.591-1.275) | 0.476 |
|  |  |  | CRS III | 1.348 (0.881-2.063) | 0.169 |

M0, crude model; M1, adjusted for age and sex; M2, adjusted for age, sex, years of education, and smoking status; M3, adjusted for age, sex, years of education, smoking status, household income, diet quality, and physical activity.

Statistically significant p-values are bolded.

**Table S2.** Sensitivity analyses for men and women in prevalent MetS**.**

|  |  |  |  |  |  |
| --- | --- | --- | --- | --- | --- |
| Sex | **Cases / total n** | **Model** |  | **OR (95% CI)** | **p-value** |
| Men | 898 / 1753 | M0 | CRS II | 1.083 (0.861-1.362) | 0.498 |
|  |  |  | CRS III | 1.434 (1.109-1.856) | **0.006** |
|  |  | M1 | CRS II | 1.110 (0.873-1.412) | 0.394 |
|  |  |  | CRS III | 1.323 (1.011-1.734) | **0.042** |
|  |  | M2 | CRS II | 1.103 (0.866-1.405) | 0.429 |
|  |  |  | CRS III | 1.346 (1.026-1.768) | **0.032** |
|  |  | M3 | CRS II | 1.095 (0.853-1.406) | 0.478 |
|  |  |  | CRS III | 1.318 (0.995-1.745) | 0.054 |
| Women | 800 / 1976 | M0 | CRS II | 1.122 (0.905-1.393) | 0.294 |
|  |  |  | CRS III | 1.334 (1.033-1.724) | **0.027** |
|  |  | M1 | CRS II | 1.272 (1.007-1.610) | **0.044** |
|  |  |  | CRS III | 1.301 (0.985-1.718) | 0.064 |
|  |  | M2 | CRS II | 1.320 (1.041-1.677) | **0.022** |
|  |  |  | CRS III | 1.326 (0.999-1.761) | 0.051 |
|  |  | M3 | CRS II | 1.330 (1.039-1.706) | **0.024** |
|  |  |  | CRS III | 1.369 (1.021-1.837) | **0.036** |

M0, crude model; M1, adjusted for age and sex; M2, adjusted for age, sex, years of education, and smoking status; M3, adjusted for age, sex, years of education, smoking status, household income, diet quality, and physical activity.

Statistically significant p-values are bolded.

**Table S3.** Sensitivity analyses for smoking status in prevalent MetS**.**

|  |  |  |  |  |  |
| --- | --- | --- | --- | --- | --- |
| Smoking status | **Cases / total n** | **Model** |  | **OR (95% CI)** | **p-value** |
| Smokers | 315 / 725 | M0 | CRS II | 1.295 (0.927-1.814) | 0.131 |
|  |  |  | CRS III | 1.764 (1.158-1.694) | **0.008** |
|  |  | M1 | CRS II | 1.299 (0.916-1.845) | 0.143 |
|  |  |  | CRS III | 1.640 (1.056-2.554) | **0.028** |
|  |  | M2 | CRS II | 1.309 (0.922-1.862) | 0.133 |
|  |  |  | CRS III | 1.629 (1.046-2.545) | **0.031** |
|  |  | M3 | CRS II | 1.231 (0.858-1.769) | 0.260 |
|  |  |  | CRS III | 1.564 (0.992-2.470) | 0.054 |
| Non-smokers | 1374 / 2988 | M0 | CRS II | 1.036 (0.868-1.238) | 0.693 |
|  |  |  | CRS III | 1.317 (1.077-1.611) | **0.007** |
|  |  | M1 | CRS II | 1.166 (0.963-1.413) | 0.117 |
|  |  |  | CRS III | 1.257 (1.012-1.562) | **0.039** |
|  |  | M2 | CRS II | 1.173 (0.967-1.423) | 0.107 |
|  |  |  | CRS III | 1.272 (1.022-1.584) | **0.031** |
|  |  | M3 | CRS II | 1.181 (0.966-1.444) | 0.105 |
|  |  |  | CRS III | 1.299 (1.036-1.630) | **0.023** |

M0, crude model; M1, adjusted for age and sex; M2, adjusted for age, sex, years of education, and smoking status; M3, adjusted for age, sex, years of education, smoking status, household income, diet quality, and physical activity.

Statistically significant p-values are bolded.

**Table S4.** Mediation analyses for incident MetS**.**

|  |  |  |  |  |  |
| --- | --- | --- | --- | --- | --- |
| CRP (log_10_) | **Model** |  | **Bootstrapped**  **mean coefficient** | **Bootstrapped**  **95% CI** | **Significance** |
|  | M1 | Direct effect (OR) | 1.321 | 0.979-1.792 |  |
|  |  | Indirect effect (OR) | 1.031 | 0.997-1.083 |  |
|  |  | E → M (β) | 0.106 (0.059) | -0.011-0.221 |  |
|  |  | M → O (β) | 0.287 (0.103) | 0.092-0.496 | * |
|  |  | E → O (β) | 0.278 (0.154) | -0.021-0.583 |  |
|  | M2 | Direct effect (OR) | 1.289 | 0.953-1.755 |  |
|  |  | Indirect effect (OR) | 1.036 | 1.001-1.093 | * |
|  |  | E → M (β) | 0.124 (0.059) | 0.008-0.243 | * |
|  |  | M → O (β) | 0.286 (0.108) | 0.084-0.508 | * |
|  |  | E → O (β) | 0.254 (0.154) | -0.048-0.563 |  |
|  | M3 | Direct effect (OR) | 1.288 | 0.945-1.769 |  |
|  |  | Indirect effect (OR) | 1.036 | 1.001-1.093 | * |
|  |  | E → M (β) | 0.120 (0.059) | 0.004-0.236 | * |
|  |  | M → O (β) | 0.297 (0.111) | 0.089-0.522 | * |
|  |  | E → O (β) | 0.253 (0.161) | -0.057-0.570 |  |
| HOMA-IR (log_10_) | M1 | Direct effect (OR) | 1.256 | 0.921-1.709 |  |
|  |  | Indirect effect (OR) | 1.062 | 0.989-1.155 |  |
|  |  | E → M (β) | 0.103 (0.062) | -0.019-0.222 |  |
|  |  | M → O (β) | 0.586 (0.120) | 0.362-0.832 | * |
|  |  | E → O (β) | 0.228 (0.158) | -0.082-0.536 |  |
|  | M2 | Direct effect (OR) | 1.254 | 0.921-1.715 |  |
|  |  | Indirect effect (OR) | 1.056 | 0.979-1.149 |  |
|  |  | E → M (β) | 0.090 (0.063) | -0.036-0.212 |  |
|  |  | M → O (β) | 0.603 (0.121) | 0.377-0.849 | * |
|  |  | E → O (β) | 0.226 (0.158) | -0.083-0.539 |  |
|  | M3 | Direct effect (OR) | 1.275 | 0.929-1.769 |  |
|  |  | Indirect effect (OR) | 1.046 | 0.962-1.145 |  |
|  |  | E → M (β) | 0.068 (0.064) | -0.059-0.193 |  |
|  |  | M → O (β) | 0.659 (0.129) | 0.417-0.929 | * |
|  |  | E → O (β) | 0.243 (0.165) | -0.073-0.570 |  |

M1, adjusted for age and sex; M2, adjusted for age, sex, years of education, and smoking status; M3, adjusted for age, sex, years of education, smoking status, household income, diet quality, and physical activity.

Abbreviations: E, exposure; M, mediator; O, outcome

Statistically significant results are indicated with asterisk.

**Figure S1.** Flow chart of the study.
